# Capability, Opportunity, and Motivation Model for Behavior Change in People With Asthma: Protocol for a Cross-Sectional Study

**DOI:** 10.2196/44710

**Published:** 2023-07-06

**Authors:** Alice Munns, Laura Wiffen, Thomas Brown, Alessandra Fasulo, Milan Chauhan, Leon D'Cruz, Daphne Kaklamanou, Anoop J Chauhan

**Affiliations:** 1 Portsmouth Hospitals University National Health Service Trust Portsmouth United Kingdom; 2 Faculty of Science and Health University of Portsmouth Portsmouth United Kingdom

**Keywords:** adherence, asthma, behavioral barriers, psychological barriers, capability, opportunity, and motivation model of behavior change, COM-B, medication, theoretical domains framework

## Abstract

**Background:**

Asthma is a common lung condition that cannot be cured, but it can usually be effectively managed using available treatments. Despite this, it is widely acknowledged that 70% of patients do not adhere to their asthma treatment. Personalizing treatment by providing the most appropriate interventions based on the patient’s psychological or behavioral needs produces successful behavior change. However, health care providers have limited available resources to deliver a patient-centered approach for their psychological or behavioral needs, resulting in a current one-size-fits-all strategy due to the nonfeasible nature of existing surveys. The solution would be to provide health care professionals with a clinically feasible questionnaire that identifies the patient’s personal psychological and behavioral factors related to adherence.

**Objective:**

We aim to apply the capability, opportunity, and motivation model of behavior change (COM-B) questionnaire to detect a patient’s perceived psychological and behavioral barriers to adherence. Additionally, we aim to explore the key psychological and behavioral barriers indicated by the COM-B questionnaire and adherence to treatment in patients with confirmed asthma with heterogeneous severity. Exploratory objectives will include a focus on the associations between the COM-B questionnaire responses and asthma phenotype, including clinical, biological, psychosocial, and behavioral components.

**Methods:**

In a single visit, participants visiting Portsmouth Hospital’s asthma clinic with a diagnosis of asthma will be asked to complete a 20-minute questionnaire on an iPad about their psychological and behavioral barriers following the theoretical domains framework and capability, opportunity, and motivation model. Participants’ data are routinely collected, including demographics, asthma characteristics, asthma control, asthma quality of life, and medication regime, which will be recorded on an electronic data capture form.

**Results:**

The study is already underway, and it is anticipated that the results will be available by early 2023.

**Conclusions:**

The COM-B asthma study will investigate an easily accessible theory-based tool (a questionnaire) for identifying psychological and behavioral barriers in patients with asthma who are not adhering to their treatment. This will provide useful information on the behavioral barriers to asthma adherence and whether or not a questionnaire can be used to identify these needs. The highlighted barriers will improve health care professionals’ knowledge of this important subject, and participants will benefit from the study by removing their barriers. Overall, this will enable health care professionals to use effective individualized interventions to support improved medication adherence while also recognizing and meeting the psychological needs of patients with asthma.

**Trial Registration:**

ClinicalTrials.gov NCT05643924; https://clinicaltrials.gov/ct2/show/NCT05643924

**International Registered Report Identifier (IRRID):**

DERR1-10.2196/44710

## Introduction

### Background

Asthma is a common lung condition affecting over 5.4 million people in the United Kingdom. For some people, it can affect daily activities and lead to life-threatening consequences [1]. Asthma cannot be cured, but it can usually be effectively controlled with available treatments, provided they are taken as recommended [1]; however, it is well recognized that up to 70% of patients do not take their asthma treatments as recommended (ie, they are nonadherent) [2]. This is perhaps not unexpected in the context of a chronic condition requiring daily treatments, which must be taken even when the person feels well [3]. People may also have concerns about using long-term medicines (including steroid inhalers), or there may be other psychological or behavioral barriers to adherence [4]. Nonadherence to asthma treatments results in poor asthma control with persistent asthma symptoms, more frequent asthma attacks, and greater impairment in quality of life [5]. There are also significant economic implications associated with “wasted” medications and additional health care requirements, such as doctors’ appointments and visits to the accident and emergency department, which contribute to the overall cost of asthma care in the United Kingdom, exceeding £1 billion (1£=US $1.103) per year [6].

Health care professionals play an important role in engaging and educating patients about the importance of their asthma treatments, monitoring asthma control, and identifying whether a patient is adherent or not [7]. Adherence is a complex process for the patient, who must be able to start prescribed medications, take the medications as prescribed (correct dose, inhalation technique, and frequency), and be persistent (obtaining refills to maintain adherence over time) [8]. There are limited resources available in a busy health care setting to ensure a complete understanding of each individual’s psychological and behavioral barriers, with a one-size-fits-all approach obtaining limited success [9]. As such, there is a persistent unmet need for a tool that can effectively identify these barriers to adherence in a clinical setting, allowing health care professionals to provide effective personalized treatments for nonadherence in patients with asthma.

Asthma adherence research has been able to identify population-specific psychological or behavioral needs, including (1) partnership between patient and health care professional [8], (2) issues around medication, (3) education about asthma and its management, (4) health beliefs, (5) self-management interventions, (6) comorbidities, (7) mood disorders and anxiety, (8) social support, (9) nonpharmacological methods, (10) access to health care, and (11) professional factors. However, in the clinical field, such knowledge is relatively limited, with findings only showing that people with asthma are vulnerable to adherence problems and that targeted adherence interventions to facilitate population-specific barriers are not always effective. Thus, beyond identifying the vulnerability to nonadherence and targeting known barriers, a patient’s personal psychological needs must be understood with in-depth and detailed insight. In addition, there is a need to establish which techniques, tools, or services are likely to successfully promote individualized ways to encourage behavioral change and adherence optimization.

In a clinical setting, health care professionals only have the resources to identify whether a patient is adherent or nonadherent to treatment; as a result, they are responsible for engaging and educating patients about the importance of their asthma treatments and monitoring asthma control. This assessment does not consider the individual barriers to each patient’s adherence, and the one-size-fits-all strategy is not optimum. However, because the patient’s complete picture is not presented (due to lack of feasibility, such as health care professionals’ time), health care professionals lack the resources to empower the patient to address their psychological needs. Therefore, a practical resource is needed for the health care provider to receive a 360-degree picture to help personalize each patient’s treatment effectively.

### The Theory

It is important that the psychological needs of patients can be easily assessed to allow for the patient’s personal needs to be understood, promoted, improved, and supported [10]. When focusing on adherence behavior, the currently available tools are questionnaires, but these are long and complex to complete. The capability, opportunity, and motivation model of behavior change (COM-B) model is a new questionnaire that is shorter and easier for patients to complete [11]. It involves 3 key components: capability, opportunity, and motivation [12]. This theoretical approach has not been used extensively for people with asthma. However, COM-B is meant to be comprehensive, concise, and applicable to all behaviors. The COM-B is designed to serve as a starting point for selecting interventions that are most likely to be beneficial, and particular interventions to address each component have been identified [13].

**Figure 1:**
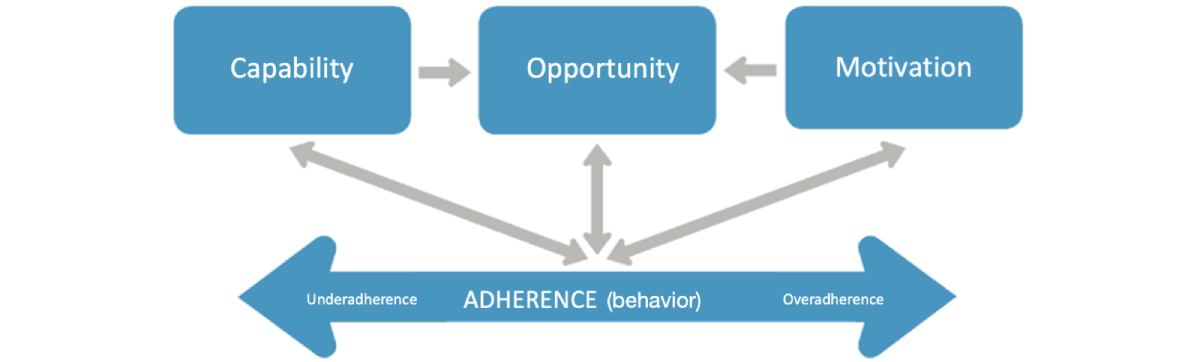
Application of the capability, opportunity, and motivation model of behavior change to adherence [16].

**Figure 2:**
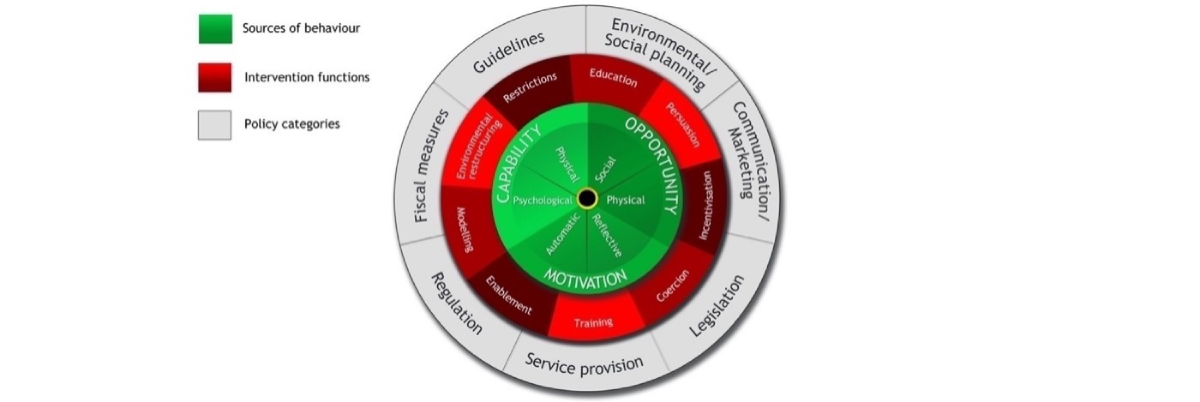
The behavioral change wheel, capability, opportunity, and motivation model of behavior change at the center [15].

The model suggests that at any time, a particular behavior will occur when the person concerned has the capability and opportunity to engage in the behavior and is more motivated to enact the behavior over other competing behaviors. Having high COM would lead to the successful performance of behavior (see Figure 1 for a visual representation of the COM-B for adherence [14]). COM-B is designed to provide an overarching model that captures all the factors known to influence behavior change: physical capability, psychological capability, physical opportunity, social opportunity, reflective motivation, and automatic motivation [15]. Its origin can be found in the 14 domains of the theoretical domains framework (TDF: see Table 1). The TDF is an overall theoretical framework consisting of 14 domains that integrate components from numerous health behavior change theories; hence, these 14 domains are regarded as essential aspects of behavior change in health psychology. The TDF can be used to help discover the elements influencing a certain behavior without overlooking key factors. As a result, the COM-B model comprises components that are hypothesized to drive behavior. Additionally, the COM-B can be placed at the heart of the behavioral change wheel (see Figure 2); thus, this framework can be used as a mapping system [16].

Despite the COM-B model being widespread, there is only one generic self-evaluation questionnaire to assess people’s perceptions of capabilities, opportunities, and motivations intended for use in multiple behaviors and a range of diverse populations [13]. Although there was evidence of this questionnaire’s acceptance, validity, and reliability, this questionnaire has not been used with patients in a medical setting. As a result, this study will be able to further demonstrate the questionnaire’s usefulness and reliability.

**Table 1:** Capability, opportunity, and motivation model of behavior change and the 11 domains of the theoretical domains framework mapped out [10].

Components of the capability, opportunity, and motivation model of behavior change			Domains in the theoretical domains framework
**Capability**			
	Psychological	Knowledge Skills Memory, attention, and decision processes Behavioral regulation	
	Physical	Skills	
**Opportunity**			
	Social	Social influences	
	Physical	Environmental context and resources	
**Motivation**			
	Reflective	Social or professional role and identity Beliefs about capabilities Optimism Beliefs about consequences Intentions Goals	
	Automatic	Social or professional role and identity Optimism Reinforcement Emotion	

### Overview

This study will assess whether the brief COM-B questionnaire can identify the same psychological and behavioral barriers as the current longer questionnaires (barriers to medication adherence questionnaire) and therefore assess the COM-B questionnaire’s ability to identify any important factors that may need to be addressed to support patients to take their asthma treatments as recommended.

We will also see whether there are any links between the type and severity of asthma that someone has, their other medical problems, how much treatment they have to take each day, and the COM-B questionnaire responses. This may help us better identify people who may struggle to take their medications properly and why. Again, we will use information routinely collected in the clinic, including lung function test results and medication prescription reviews, to do this.

## Methods

### Overview

This study will employ a cross-sectional survey design focusing on adherence to asthma treatment. It will be delivered through REDCap, a web-based platform. Participants will be recruited when attending their routine clinical review with the asthma service or while an inpatient at Queen Alexandra Hospital in Portsmouth, United Kingdom. Routinely collected patient data, including demographics, asthma characteristics, asthma control, and medication regime that is already collected as part of their clinical review, will be recorded on an electronic data capture form (e-CRF) through the REDCap platform. They will then be asked to complete the series of questionnaires, all completed on the web. Patients will also be provided with a paper copy of each questionnaire. When the questionnaire is completed, the user will be provided with debriefing information.

Participants will be asked to complete a set of questionnaires that will assess the following:

1. Psychological and behavioral barriers that may affect the participant being able to take their asthma treatments as recommended: the brief COM-B questionnaire (which is being validated as part of this study) and the 61-item Theoretical Domain Framework Questionnaire.
2. Their asthma symptoms and how well controlled their asthma is (Asthma Control Questionnaire [ACQ-6]), the impact their asthma has on their quality of life (Mini Asthma Quality of Life Questionnaire), and the level of anxiety or depression they may be experiencing (Hospital Anxiety and Depression Scale). All of these questionnaires are used routinely at clinic reviews.
3. Their use of technology at home and any challenges they face with this: Digital Readiness Questionnaire.

The COVID-19 pandemic has changed the way people with asthma access their care with a move to remote consultations and questionnaire completion. The COM-B questionnaire may well be suitable for this approach. However, it is essential to assess digital readiness in this population to understand whether this is feasible and inform the design of future studies if the COM-B questionnaire proves to be successful.

It should not take more than 20 minutes to complete. The questionnaires are all validated and approval has been granted for their use in this study.

### Eligibility Criteria

Participants will be drawn from a range of adults with asthma who take regular medication for their condition. The inclusion and exclusion criteria are presented in Textbox 1.

**Inclusion and exclusion criteria of the study participants.**

**Inclusion criteria**

- Aged 18 years or above
- Willing and able to give informed consent for participation in the study
- Diagnosing of asthma confirmed by a health care professional and requiring maintenance asthma treatment, including an inhaled corticosteroid.

**Exclusion criteria**

- Unable to comprehend the study and provide informed consent, for example, insufficient command of English in the absence of someone who can adequately interpret.

### Sampling and Sample Size

A power calculation was conducted to determine the sample size, using the 6 components of the COM-B model that are hypothesized to drive behavior, namely physical capability, psychological capability, physical opportunity, social opportunity, reflective motivation, and automatic motivation. Using the G-power analysis for 6 predictors, a minimum of 146 participants are needed. The G-power analysis was run using SPSS to offer a sensitivity power analysis of the effect size that this sample size was sensitive to detect.

As a result, to detect a standardized effect size of Cohen ​*d*=0.43 with 80% power (α =.05, 2-tailed), G*Power suggests we would need 146 participants in independent tests. A paired sample *t* test with 30 participants would be sensitive to the effects of Cohen *d*=0.53 with 80% power (α=.05, 2-tailed). This means the study would not reliably detect effects smaller than Cohen *d*=0.53. To detect a Pearson correlation coefficient of ρ=.21 with 80% power (α=.05, 2-tailed), G*Power suggests we would need 146 participants.

### Recruitment

All people with asthma who are under the care of the asthma service at Portsmouth Hospitals University National Health Service (NHS) Trust will be invited to participate in this study. This will include recruitment from primary care sites. In addition, both outpatients and inpatients will be approached and invited to participate.

### Study Assessment

This section describes the information that will be recorded in the participant’s RedCap report, which will be anonymized using the participant’s unique study number.

#### Participant Clinical Characteristics

The following information is routinely collected and used as part of standard care and following consent and recruitment to the study will be transcribed from a participant’s clinical care records and will be recorded on the e-CRF.

#### Patient Details and Demographics

1. Age, gender, ethnicity, school or work status, educational level
2. Smoking status and pack-year smoking history
3. Asthma severity is defined using British Thoracic Society stages
4. New or established patients to the asthma service
5. Medical comorbidities

#### Asthma Characteristics

1. Age when asthma diagnosis was made
2. Spirometry—this will either be captured as part of their routine clinic visit or can be historic (within the last 12 months)
3. Type 2 (T2) biomarkers: peripheral blood eosinophil count and fractional exhaled nitric oxide (FeNO)—this will either be captured as part of their routine clinic visit or can be historic (within the last 12 months)
4. Number of exacerbations in the preceding 12 months (number of courses of steroids, number of emergency department attendances or hospital admissions)
5. Current asthma treatments

#### Adherence Assessment

1. Medicines possession ratio (MPR)—this measurement will consider the past 12 months of the participant’s adherence. Adherence will be defined as an MPR ≥80%
2. Advanced measures of adherence (such as FeNO suppression) where available

#### Complexity of Medication Regime

1. Total number of medications taken each day
2. Number of doses of inhaled medications per day

#### Questionnaires Collected

The asthma COM-B questionnaire [13] measure will be responsible for identifying one’s capabilities, opportunities, and motivations regarding asthma medication adherence. In the questionnaire, these measures will be presented as individual questions, forming 6 matrix tables.

**Figure 3:**
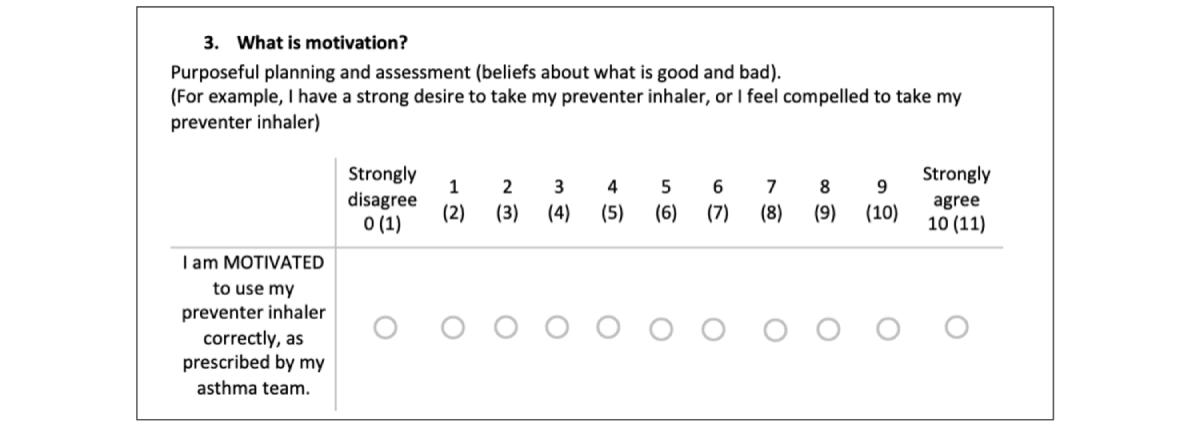
Items 1 of 6 from the adapted capability, opportunity, and motivation model of behavior change questionnaire to target known drivers of adherence in people with asthma. This figure was adapted from Keyworth C et al [17] which is published under Creative Commons Attribution 4.0 International License [20].

This questionnaire will be brief, with 6 items asking them to rate their understanding of their psychological and physical capacities, opportunities, and motivations for adhering to asthma treatment on a Likert scale (see Figure 3, for example items from the questionnaire adapted from Keyworth et al’s [17] COM-B measure). This tool would follow the structure, formatting, and length of other questionnaires given to patients during their initial assessments. As a result, it will be easily integrated into the health care system, providing health care providers with immediate responses to patients’ psychological needs and potentially getting a better understanding when meeting with the patient.

The ACQ [15,18] consists of 6 items covering day and nighttime symptoms, activity limitations, and rescue bronchodilator use. A score of ≥1.5 reflects poor asthma control.

The Mini Asthma Quality Of Life Questionnaire standardized version [16,19] measures four domains: symptoms, activity limitation, emotional function, and environmental stimuli.

#### Adherence Measures

The 61-item Theoretical Domain Framework Questionnaire [21,22] will determine the individual’s behavior in terms of the theoretical domains framework (see Table 1).

The Test Of Adherence To Inhalers [23,24] is a 12-item questionnaire designed to assess adherence to inhalers in patients with asthma.

#### Digital Readiness Measure

The Digital Readiness Scale [17] was followed to investigate the effectiveness of using technology tools for medical practices in the NHS. This scale will be adapted to 6 items assessing participants’ use of digital tools, interest in digital tools, and challenges faced by participants while using digital tools [25].

### Statistical Analysis

#### Data Exclusion

Participants are free to withdraw from the study, and this will not affect their treatment. Participants will consent at the start of the survey to allow their anonymized study data to be retained and analyzed by the study team if they withdraw from the study. Withdrawn participants will not be replaced.

Data will be analyzed once 146 participants have completed the questionnaire. All data will be handled within the general data protection regulations conditions, which came into force in May 2018.

Once a survey is completed, it is locked, and time-stamped so the survey responses cannot be edited further. Required fields are specified on the survey form, and partial or incomplete responses will not limit the survey response. Once the survey response limit is reached, the public URL will redirect to a message notifying that the survey is closed.

#### Data Management

All data collection will be completed on the web. The research team will use the Redcap electronic cloud solution to store and manage all consent and study data. The cloud services are NHS compliant and approved for use in surveys.

Data will be stored on a secure password-protected electronic research database hosted on a secure NHS server with appropriate backup systems, antivirus software and security protocols compliant with NHS information governance standards. Data entered into the electronic database will be accessible only by a restricted number of research personnel listed in the delegation log of the survey master file.

There will be a survey master file with a delegation log of approved users in the file. Before anyone can use the database, they will have to be included in this list, with the approval of the chief investigator. The chief investigator and survey coordinator will have access to all research data collected in this study to perform the data analyses required.

A data manager will perform regular data quality control checks, and reporting and statistics will be performed using tools built into the survey distribution platform. A comprehensive audit log will be maintained throughout the study and exported and stored in the survey master file on study closer.

##### Overview

Patient scores for the behavioral components of physical capability, psychological capability, social and physical opportunity, and reflective and automatic motivation will be tabulated, so that adherence and asthma characteristics can be recorded for each behavioral source against predefined behavioral change techniques based on the behavioral change wheel [12].

##### Summary Statistics

Demographics or baseline characteristics of each participant will be produced, as well as summaries for all participants combined. Normally distributed continuous variables will be summarized by the mean to characterize the population. Nonnormally distributed variables will be summarized by the median to characterize the population.

##### Primary Analysis

The primary analysis will comprise the correlation of outcomes from the brief COM-B questionnaire and the TDF questionnaire, as well as the Spearman rank correlation coefficients (ρ; strength of the relationship) and a hierarchical multiple regression analysis. For example, the capability result of the questionnaire will be compared to knowledge, skills, memory attention and decision processes, behavioral regulation, and skills. In addition, some exploratory analysis between the variables will be conducted—this will be used to derive the content validity of the brief COM-B questionnaire.

##### Secondary Analysis

Descriptive statistics will be used to illustrate levels of asthma control, medication adherence, and perceptions of capabilities, opportunities, and motivations. One-sample *t* tests, within participants’ ANOVA and deviation contrasts will be used to explore differences between levels of capabilities, opportunities, and motivations regarding adhering to their asthma medication programs.

For each capability, opportunity, and motivation variable, separate cluster analysis models will be used to examine associations between the COM-B with levels of adherence measured by MPR and FeNO suppression to explore the key psychological and behavioral barriers indicated by the COM-B questionnaire and methods of determining adherence in a clinical setting.

Multiple regressions will be conducted to test the levels of adherence. In addition, other variables such as demographics and asthma characteristics will be taken into consideration.

##### Exploratory Analysis

Network analysis between variables including (but not limited to):

1. Type of asthma (T2 or non-T2 defined by the presence of a T2 inflammatory signal assessed using FeNO and peripheral blood eosinophil count)
2. Asthma severity using British Thoracic Society or Scottish Intercollegiate Guidelines Network (SIGN) stage
3. Level of asthma control assessed using the ACQ6 questionnaire (a score of ≥1.5 reflects poor asthma control)
4. Quality of life measured using the mini-asthma quality-of-life questionnaire score
5. Number of comorbidities
6. Complexity of treatment (number of inhaled doses per day, total number of medications taken each day)
7. Informative analysis of digital readiness will be performed by summarizing all participants combined responses, reported in percentages

##### Procedure for Missing, Unused, or Spurious Data

There are no plans for multiple imputations or other corrections for missing data.

### Ethical Considerations

#### Statement of Compliance

All staff working on this study will hold evidence of good clinical practice training before undertaking any responsibilities, and all staff working within the research study team are also part of the respiratory clinical care team and are involved in PhD supervision. Written informed consent will be obtained from all participants after an adequate explanation of the aims, methods, and anticipated benefit of the study using the participant information sheet. A signed copy of the consent form will be given to the participant, and copies will be filed in the study master file and the participants’ medical notes.

Participants’ anonymity will be maintained throughout by identification on a password-protected electronic database and e-CRFs only by initials and participant ID number. All documents will be stored securely and only accessible by study staff and authorized personnel; the study will comply with the Data Protection Act.

#### Potential Benefits or Risks of Study Participation

This study is a low-risk questionnaire study; however, any risks to the participants have been carefully considered—any participants who were observed struggling with the psychological aspect of the questionnaire or the questionnaire raised further issues patients will have these errors highlighted and will be informed with the correct health care professional to look after their care. This is a beneficial outcome for participants’ self-care and management and ensures that they obtain the correct level of care.

This study analyzes a wide variety of behavioral barriers and will help provide a clear picture of the psychological needs that are associated with asthma adherence. Understanding this information is important in the context of managing lung conditions, from treating patients in primary care through to feeding this information back to intervention designers.

#### Other Ethical Considerations

The study will not be initiated before the protocol, and the survey’s material such as the consent forms, survey answers, and participant information sheets have received favorable opinions from the health research authority and the respective NHS research and development (R&D) departments. Should an amendment be made that requires Research Ethics Committee (REC) approval, as defined by REC as a substantial amendment, the changes will not be instituted until the amendment has been reviewed and received approval or favorable opinion from the REC and R&D departments. However, a protocol amendment intended to eliminate an apparent immediate hazard to participants may be implemented immediately, providing that the REC is notified as soon as possible, and approval is requested. Other amendments will be implemented under the direction of the REC.

All participants will have adequate time to consider participation in the study, as per good clinical practice guidelines.

Participants who are already enrolled in other research surveys will be invited and allowed to participate in the study if they so wish.

#### Patient Public Involvement Process

Patients having first-hand knowledge of coping with chronic respiratory disease were sought out for patient involvement in this study. We have spoken with our respiratory patient representatives in person, over the phone, and via email. These individuals have participated in earlier research projects and have lived with respiratory problems. To ensure that we address the issues that are pertinent to persons with airway disorders, they helped define the main questions and establish our study objectives. To reduce waiting times for patients who decided to participate in the study, assistance was also sought with the design of participant recruitment and the implementation of the study within a typical clinical visit. Additionally, they contributed to creating the participation information sheet and cowrote the lay summary.

## Results

The study is already underway, and it is anticipated that the results will be available by early 2023.

## Discussion

The COM-B asthma study will investigate an easily accessible theory-based tool (a questionnaire) for identifying psychological and behavioral barriers in patients with asthma who are not adhering to their treatment. This will provide useful information on the behavioral barriers to asthma adherence and whether or not a questionnaire can be used to identify these needs. The highlighted barriers will increase health care professionals’ knowledge of this important subject, allowing for practice change and better information for interventions to remove those barriers. Overall, this will enable health care professionals to use effective individualized interventions to support improved medication adherence while also recognizing and meeting the psychological needs of patients with asthma.

## Abbreviations

ACQ: asthma control questionnaire
COM-B: capability, opportunity, and motivation model of behavior change
e-CRF: electronic data capture form
FeNO: fractional exhaled nitric oxide
MPR: medicines possession ratio
NHS: National Health Service
R&D: research and development
REC: Research Ethics Committee
SIGN: Scottish Intercollegiate Guidelines Network
T2: type 2
TDF: theoretical domains framework

## Data Availability

The data sets generated or analyzed during the current study are available from the corresponding author on reasonable request.
